# Reported Dietary Intake, Disparity between the Reported Consumption and the Level Needed for Adequacy and Food Sources of Calcium, Phosphorus, Magnesium and Vitamin D in the Spanish Population: Findings from the ANIBES Study †

**DOI:** 10.3390/nu9020168

**Published:** 2017-02-21

**Authors:** Josune Olza, Javier Aranceta-Bartrina, Marcela González-Gross, Rosa M. Ortega, Lluis Serra-Majem, Gregorio Varela-Moreiras, Ángel Gil

**Affiliations:** 1 Department of Biochemistry and Molecular Biology II, Institute of Nutrition and Food Sciences, University of Granada, Campus de la Salud, Avda. del Conocimiento, Armilla, 18100 Granada, Spain; jolza@ugr.es; 2 Instituto de Investigación Biosanitaria ibs.GRANADA, 18012 Granada, Spain; 3 CIBEROBN, Biomedical Research Networking Center for Physiopathology of Obesity and Nutrition, Carlos III Health Institute, 28029 Madrid, Spain; javieraranceta@hotmail.com (J.A.-B.); marcela.gonzalez.gross@upm.es (M.G.-G.); lluis.serra@ulpgc.es (L.S.-M.); 4 Department of Preventive Medicine and Public Health, University of Navarra, c/Irunlarrea 1, 31008 Pamplona, Spain; 5 ImFINE Research Group, Department of Health and Human Performance, Technical University of Madrid, c/Martín Fierro 7, 28040 Madrid, Spain; 6 Department of Nutrition, Faculty of Pharmacy, Madrid Complutense University, Plaza Ramón y Cajal s/n, 28040 Madrid, Spain; rortega@ucm.es; 7 Research Institute of Biomedical and Health Sciences, University of Las Palmas de Gran Canaria, Faculty of Health Science, c/Doctor Pasteur s/n Trasera del Hospital, Las Palmas de Gran Canaria, 35016 Las Palmas, Spain; 8 Spanish Nutrition Foundation (FEN), 28010 Madrid, Spain; gvarela@ceu.es or gvarela@fen.org.es; 9 Department of Pharmaceutical and Health Sciences, Faculty of Pharmacy, CEU San Pablo University, Urb. Montepríncipe, Crta. Boadilla Km 53, Boadilla del Monte, 28668 Madrid, Spain

**Keywords:** ANIBES study, calcium, food intake, magnesium, misreporting, nutrients, phosphorus, vitamin D

## Abstract

Calcium, phosphorus, magnesium and vitamin D have important biological roles in the body, especially in bone metabolism. We aimed to study the reported intake, the disparity between the reported consumption and the level needed for adequacy and food sources of these four nutrients in the Spanish population. We assessed the reported intake for both, general population and plausible reporters. Results were extracted from the ANIBES survey, *n* = 2009. Three-day dietary reported intake data were obtained and misreporting was assessed according to the European Food Safety Authority (EFSA). Mean ± SEM (range) total reported consumption of calcium, phosphorus, magnesium, and vitamin D for the whole population were 698 ± 7 mg/day (71–2551 mg/day), 1176 ± 8 mg/day, (331–4429 mg/day), 222 ± 2 mg/day (73–782 mg/day), and 4.4 ± 0.1 µg/day (0.0–74.2 µg/day), respectively. In the whole group, 76% and 66%; 79% and 72%; and 94% and 93% of the population had reported intakes below 80% of the national and European recommended daily intakes for calcium, magnesium and vitamin D, respectively; these percentages were over 40% when the plausible reporters were analysed separately. The main food sources were milk and dairy products for calcium and phosphorus, cereals and grains for magnesium and fish for vitamin D. In conclusion, there is an important percentage of the Spanish ANIBES population not meeting the recommended intakes for calcium, magnesium and vitamin D.

## 1. Introduction

The decline of essential nutrients deficiencies over the past century along with the improvement in the treatment of infectious diseases has contributed to the increase in population life expectancy [1]. However, in recent years, rates of nutrition-related chronic diseases, such as obesity, cardiovascular diseases and type 2 diabetes mellitus (T2DM) have increased, dwindling the quality of life of the population [1,2,3].

Studying the nutritional situation of the population as well as lifestyle habits is fundamental to design national guidelines and public policies. Calcium, phosphorus, magnesium and vitamin D participate mainly in bone development and maintenance, but they also have other relevant biological roles [4,5]. Inadequate intake and low nutritional status of calcium, magnesium and vitamin D, are well documented in many populations worldwide [5,6,7]. Some studies have demonstrated that the consumption of low-fat milk and dairy products is inversely associated with the risk of hypertension [8,9]. Likewise, several studies have shown that a higher consumption of milk and dairy products rich in calcium is associated with lower incidence of T2DM [10,11,12,13]. Nevertheless, it is important to highlight that the diet is not the only factor that increases or decreases the risk of developing chronic diseases; there are other factors as the genetic background and lifestyle habits that also contribute to the risk and prevalence of these diseases.

Calcium is the main mineral involved in the structural integrity of the organism; in addition to its role in the formation and maintenance of bones and teeth, it is essential for many metabolic processes, specifically, as cell second messenger. Besides, it is necessary for the maintenance of the blood coagulation. Calcium has the highest requirements among all minerals [14]. Phosphorus is also found in the mineral structure of bones and teeth and in soft tissues where it participates mainly in phosphorylation processes and acid-base equilibrium. Its deficiency is not common as it is present in most foods and its absorption is relatively high [15]. Magnesium is the second most abundant intracellular cation, 70% of this mineral is in the skeleton and the rest in the cells. This mineral participates in more that 300 enzyme reactions and has similar functions as calcium, such as muscular contraction, gland secretions and nerve transmission, among others [16]. Magnesium absorption is approx. 50%, and in general population its deficiency is not uncommon, as its intake has diminished over the years [17].

Vitamin D, a fat-soluble vitamin, is the main regulator of serum calcium and phosphorus homoeostasis. It also participates in cell differentiation and proliferation and has effects on the immune and nervous system responses [18]. Its deficiency is prevalent worldwide, but proportions vary among world regions [19]. The main source of vitamin D is endogenous; that is to say, the daily exposure to the sun. Without vitamin D food fortification, the dietary intake of this vitamin is low [20].

National diet and nutritional survey is the most used tool to assess the diet, nutrient reported intake and nutritional reported status of the population. The data collected in the surveys are mostly based on subjects self-reporting. As this method is indirect and has a pseudo-quantitative nature, the surveys frequently report data that do not represent the habitual intake of the studied population and estimate energy intakes (EI) that are not plausible physiologically [21]. In this respect, EFSA has published a protocol that has a harmonised approach to identify misreporting [22]. As we do in the present article, EFSA suggests that the data should be reported for the whole population as well as divided into plausible and non-plausible reporters. Additionally, it is important to mention that in recent years there has been an open discussion about the validity of the use of Memory-Based Dietary Assessment Methods (M-BMs) to collect dietary intake data [23]. Some authors believe that the use of 24-h recall is inappropriate to calculate EI and that these data are inadmissible in scientific research and for the formulation of national dietary guidelines, while others refute these statements [23,24]. It is well known that currently there is not a gold standard method to collect nutritional intake data and there is a need for an accurate scientific methodology. In this respect, some modest improvements have been made in the development of more objective tools to measure EI as digital photography or chewing and swallowing monitors; however, more research is needed to develop other objective tools [25]. In the recent years, EFSA has published the “Guidance on the EU Menu methodology”, which is a guidance document developed to facilitate the collection of more harmonised food consumption data from all EU Member States [26] and the present study is based on these guidelines. An additional aspect to consider is that the estimation of usual micronutrients intake using complex sample derived data from few days dietary food records, introduce excessive intra-individual variation, and to overcome this problem different statistical procedures have been reported [27,28,29] and provide another approach to managing and analysing intake population data.

The ANIBES Spanish study aimed to evaluate energy reported intake, energy expenditure, body composition, dietary patterns and dietary quality indexes in a national representative sample of the Spanish population by using innovative methodological tools [30]. The present article analyses the disparity between the reported consumption and the level needed for adequacy of the main nutrients involved in bone metabolism (calcium, phosphorus, magnesium and vitamin D), considering the coefficients of within-person variations for different age groups, as well as the food and beverages that contribute to their sources of intake in the Spanish population. We assessed the reported intake for both, general population and plausible reporters.

## 2. Materials and Methods

The complete design, protocol, and methodology of the ANIBES study have previously been described in detail elsewhere [30,31].

### 2.1. Sample

The ANIBES study is a cross-sectional study conducted using multistage stratified sampling. The sample for the ANIBES Study was designed based on 2012 census data published by the INE (Instituto Nacional de Estadística/Spanish Bureau of Statistics) for Gender, Age, Habitat Size and Region [30]. The fieldwork was performed at 128 sampling points across Spain to guarantee better coverage and representativeness. The design of the ANIBES study aims to define a sample size that represents all individuals living in Spain, aged 9–75 years and residing in municipalities of at least 2000 inhabitants. The initial potential sample was 2634 individuals, and the final sample was 2009 individuals (2.23% error and 95.5% confidence interval). In addition, for the youngest (9–12, 13–17 and 18–24 years old) and oldest (65–75 years) age groups, a boost sample was considered in order to have at least a *n* = 200 per age group and increase the statistical power of the study (error +/−6.9%) [30]. The booster sample is the process of increasing the number of interviews for a particular subgroup within the population. This method allows achieving an adequate number of interviews to allow the analysis of population subgroups, without the expense of increasing the sample size for the whole survey. Therefore, the random sample plus booster sample comprised 2285 participants [32]. For all analyses, in this study, the entire population was used.

The ANIBES sample was comprised of 50.4% of males and 49.6% females. The sample quotas according to the following variables were: age groups (9–12, 13–17, 18–64 and 65–75 years); sex (men/women); geographical distribution (Northeast, East, Southwest, North-Central, Barcelona, Madrid, Balearic and Canary Islands); and locality size: 2000 to 30,000 inhabitants (rural population); 30,000 to 200,000 inhabitants (semi-urban population) and over 200,000 inhabitants (urban population). Additionally, other factors for sample adjustment were considered: unemployment rate, the percentage of foreigners (immigrant population), physical activity level and educational or economic level [32,33].

The fieldwork for the ANIBES study was conducted from mid-September 2013 to mid-November 2013, and two previous pilot studies were also performed. Subjects participated during two weekdays and one weekend day to equally represent all days of the week. The final protocol was approved by the Ethical Committee for Clinical Research of the Region of Madrid (Spain) [32,33].

### 2.2. Food Record and Adequacy of Reported Intake

Study participants were provided with a tablet device (Samsung Galaxy Tab 2 7.0, Samsung Electronics, Suwon, Korea). They were trained in recording information by taking photos of all food and drinks consumed during the three days of the study, both before beginning to eat and drink, and again after finishing, to record the intake and the leftovers. Additionally, a brief description of meals, recipes, brands and other information was registered using the tablet. Participants who declared or demonstrated that they were unable to use the tablet device were offered other options, such as using a digital camera and paper record and telephone interviews. In total 79% of the sample used a tablet, 12% a digital camera and 9% opted for a telephone interview. As no differences in the percentage of misreporting were found according to the type of device used to assess dietary intake, we used the measurements of the three assessment methods in the analysis. Food records were returned from the field in real time, to be coded by trained coders who were supervised by dieticians. An ad hoc central server software/database was developed for this purpose, to work in parallel with the coding and verification processes [30]. The ANIBES software received the information from the field tablets every two seconds and updated it every 30 min. The Central Server verifies the information at the individual level; food weight and intakes; food codification and the assigning weight in grammes. Finally, 189,600 inputs (ingredients) were managed from the 2009 participants, about 73 items per participant, and 24.3 food/beverages items per person/day as mean [30].

Coders attempted to match each food or drink item recorded on the tablet device with a food/portion code. For composite items, which could be split into their parts, an individual component was assigned. If an item had been recorded and there was no suitable code, or there was an insufficient detail to code the food, the entry was flagged as a query. Each food code was linked to appropriate portion size descriptors, which were then linked to the correct weight for that descriptor. If the portion size was described as weight, the weight was entered directly into the system. Where the coder could not resolve the food or portion consumed, the entry was flagged as a query for action by a researcher who had greater nutrition knowledge and experience. The dieticians-nutritionists checked and solved all the queries raised by the coders. Where portion sizes were missing, an estimate was made using the same weight if the food was consumed on another dietary day, or a portion size consistent with the participant’s frequent consumption (e.g., small, medium or large), or an age-appropriate average portion. For new products not included in the software, the nutritional information was obtained and introduced in the software. If a new food code was required, the nutrient content was entered into the database. In the case of school meals, school caterers’ information about the nutrient content and portion size of dishes were considered [30].

The quality control of the collected information was supervised by trained dieticians-nutritionists, following the protocol:

1 The same dietician-nutritionist was responsible for checking the food records included by the participant during the three-day dietary food record study.
2 The initial quality control was based on the photographs and descriptions sent by the participants, but also in the brief description that was asked before/after each meal and/or intake. Particular care was given to validate some variables such as ingredients, brands of the processed and ready-to-eat foods, portion size or culinary technique to obtain accurate information for further codification.
3 The final approval of the received information was given by a dietician-nutritionist and supervisor.

It is also of importance that the software used had an alarm system when no records from any of the different three main meals were available. At the start of the coding process, dieticians-nutritionists worked together with the coders checking the information and giving them individual feedback on their work. Where errors were found, they were corrected. All of the entries flagged as a query by the coders were categorised into different query types, such as food code or portion code not available in the used software, recipes, missing or insufficient detail to code food or portion. Final quality checking was performed using each participant’s mean energy and nutrient reported intake over the food and beverages diary record period (three days). Extreme intakes were considered from the mean, and all entries in this region were checked against the diary [30].

Food, beverage and energy and nutrient reported intakes were calculated from food reported consumption records using VD-FEN 2.1 software, a Dietary Evaluation Program from the Spanish Nutrition Foundation (FEN). The program was newly developed for the ANIBES study by the FEN and is based mainly on Spanish food composition tables [34]. Data obtained from food manufacturers and nutritional information provided on food labels were also included. A food photographic atlas was used to assist in assigning gramme weights to portion sizes. Micronutrients reference intakes for Spain [35] and Europe (EFSA) [36] were used to compare the actual reported intake with those recommended. The disparity between reported consumption and the level needed for adequacy was calculated comparing with 80% of the Spanish dietary reference value (DRV) and EFSA population reference intake (PRI) or adequate intake (AI).

### 2.3. Evaluation of Misreporting

As mentioned in the introduction section, it is well known that when the data of a nutrition survey are self-collected, people tend to underreport the consumption of food and beverages; and although over-reporting is also observed, the proportion is lower. In the present article, the EFSA recommendations to calculate misreporting was followed; the protocol proposed by EFSA is based mainly on the Goldberg [37] and Black [38,39] work. This method evaluates the reported EI (EIrep) against the presumed energy requirements. EIrep is expressed as a multiple of the mean basal metabolic rate estimated (BMRest) (from formulas), and it is compared with the presumed energy expenditure of the studied population. Then the ratio EIrep:BMRest is referred to as the physical activity levels (PAL) [22]. The PAL is established for young (≤17 years) and adults (≥18 years) in three levels, low 1.6 and 1.4; moderate 1.8 and 1.6; and vigorous 2.0 and 1.8, respectively. Additionally, the protocol indicates that analyses should be done at two levels, group and individual. The group level determines the overall bias to the reported EI, and the individual level shows the rate of under and over reporters. In the present article, we calculated the BMRest using the Schöfield equations [40]. Physical activity was assessed during face-to-face interviews with the international physical activity questionnaire (IPAQ), details are published somewhere else [41].

Misreporting cut-offs at group and individual levels for the ANIBES study are shown in Table 1. CV-WEI (coefficient of variance for energy intake within-subject) for the ANIBES population were 36.6% for children and adolescents and 41.6% for adults, respectively; and S (the factor that takes into account the variation in energy intake, BMR and PAL) for children and adolescents was 27.3 and for adults 29.6.

### 2.4. Statistical Analysis

Data are expressed as mean ± standard error of the mean (SEM), median, ranges, percentiles and percentages. Kolmogorov–Smirnoff normality test was used to check the normality of the distribution: random sample (2009 participants) and random + booster sample (2285). For those variables that did not follow the normality, appropriated non-parametric statistical tests were used for comparisons of groups. The random sample was used to show the total sample data and to compare between sexes. To compare by sex in each age group, a booster sample was included to enlarge those groups less represented in the random sample. Comparisons between groups were performed by a Student’s *t*-test for independent samples or Mann–Whitney U test to evaluate differences by sex within the whole population and within each age group. Analyses of variance (ANOVA) tests with Bonferroni correction for multiple comparisons or Kruskal–Wallis analysis were used to calculate differences among each age group. These procedures have taken into account the sampling complexity during the stratification of the study design. The level of significance was set at *p* < 0.05. Analyses were performed using IBM SPSS version 22.0 (IBM Corp., Armonk, NY, USA).

## 3. Results

### 3.1. Calcium, Phosphorus, Magnesium and Vitamin D Reported Intake and Distribution in the Whole Population

Table 2 depicts the daily reported intake levels of calcium, phosphorus, magnesium and vitamin D; Tables S1–S4 depict the percentiles distribution of each nutrient in the whole population and separately by age groups and sexes.

Men reported significantly higher consumption of calcium, phosphorus and magnesium than women in the entire population; as well as separately by age group for calcium in children, adolescents and adults, and for magnesium in adolescents, adults, and elderly. Higher reported intake of calcium was observed in children and adolescents compared with adults and elderly (*p* < 0.05). The reported consumption of phosphorus decreased, and that of vitamin D increased, both, with advancing age.

### 3.2. Calcium, Phosphorus, Magnesium and Vitamin D Reported Intake in Plausible Reporters

Table 3 shows the misreporting data. In the entire population, the plausible reporters were 543 individuals (27%), and the non-plausible reporters were 1466 (73%). The percentages of plausible reporters by age groups were: children 56%, adolescents 36%, adults 26% and elderly 22%. According to the misreporting analysis, we observed that the reported consumption of the four nutrients was significantly higher (*p* < 0.001) in the plausible reporters than in the non-plausible reporters in the whole population, as well as divided by age group, except for vitamin D in the adolescents. Likewise, there were significant differences when comparing plausible and non-plausible reporters within sexes.

### 3.3. Disparity between Reported Intake and the Level Needed for Adequacy for Calcium, Magnesium and Vitamin D in the Whole Population

Table 4 shows the percentage of the whole population and the plausible reporters that did not met the 80% of the Spanish [35] and European [36] recommended daily intake. As we can observe neither the whole population nor the plausible reporters separately met the daily intake recommendations for calcium, magnesium or vitamin D in the whole population and in the plausible reporters by gender, by age group and by gender and age group, according to the reported intake.

### 3.4. Contribution of Food Sources to Calcium, Phosphorus, Magnesium and Vitamin D Reported Intakes

The intake data were grouped into 16 food groups, 33 subgroups and 754 ingredients for in-depth analysis.

**Figure 1:**
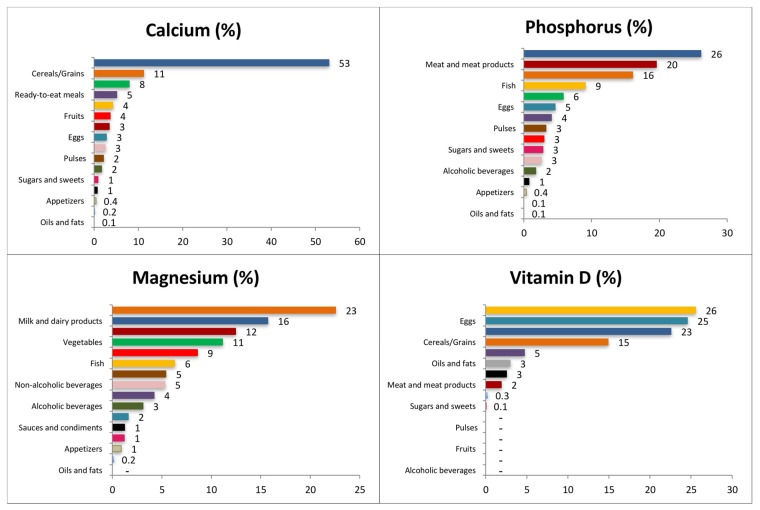
Contribution of food categories to the daily calcium, phosphorus, magnesium and vitamin D reported intake in the ANIBES Study population.

Figure 1 depicts the contribution (%) of food and beverage categories to the daily calcium, phosphorus, magnesium and vitamin D reported intake for the whole population. Tables S5–S8 show these data separately by age groups.

#### 3.4.1. Calcium

The main sources of calcium for the entire population were milk and dairy products (53.1%; this contribution was higher in children, 60.3%), cereals and grains (11.2%), and vegetables (7.9%). Cereals and grains provided higher percentages to the younger groups whereas vegetables did so to the older. Ready to eat meals (5.1%), fish (4.2%), fruits (3.6%), and meat and meat products (3.4%) complete the list to reach more than the 85% of the total reported intake of calcium. Ready to eat meals afforded a higher percentage to the adolescent’s group while fish did for the older groups.

#### 3.4.2. Phosphorus

The largest source of phosphorus for the whole population was milk and dairy products (26.1%), with a higher contribution for the children group (31%). Meat and meat products provided 19.6% and cereals and grains 16.1%. Fish contributed 9.1% and vegetables 5.8%; these last two food groups afforded less to the younger groups and more to the elderly. Eggs supplied 4.6%, and ready-to-eat meals 4.1%; both food groups contributed more to the younger groups and less to the older adults. All these groups afforded in more than 85% to the phosphorus reported intake.

#### 3.4.3. Magnesium

Cereal and grain products were the main sources of magnesium for the whole population (22.6%), contributing in higher proportions to the younger groups than to the older adults. Milk and dairy products provided 15.7% to the whole population, contributing more to the younger groups. Meat and meat products ranked third (12.5%), and vegetables fourth (11.1%); this last food group provided less among younger groups and more among older adults. Fruits supplied 8.6%, fish 6.3%, and pulses (5.4%) to the magnesium reported consumption for the whole population; these food groups contributed less to the younger groups and more to the older adults. All these groups supplied more than 80% of the total magnesium reported intake.

#### 3.4.4. Vitamin D

Fish was the main source of vitamin D (25.6%) for the whole population, although it contributed much less in younger groups than older adults. Eggs provided 24.6% and milk and dairy products 22.6%, this last food group in a higher proportion for the children group. Cereals and grains supplied 14.9% to the whole population, contributing to the largest proportion in younger groups. These four food groups contributed to more than 85% of the total daily vitamin D reported intake.

## 4. Discussion

Recent studies have demonstrated that the requirements for some minerals, namely calcium and magnesium, are not being met in all age groups, mainly because of the poor quality of the diet [5,42]. There are other micronutrients, such as vitamin D, that do not depend exclusively on food intake but also on other factors, such as endogenous synthesis [18]. Nevertheless, the diet is an important source of vitamin D, especially in places with low sun exposure. The present article shows that the reported daily dietary intakes of calcium, magnesium and vitamin D are not being met by the majority of the Spanish population included in the ANIBES study. It is important to highlight that ANIBES is the first national diet and nutrition survey in Spain, reporting nutrient intake for plausible and non-plausible reporters, based on well-harmonised procedures [22]. The ANIBES study is also based on EFSA “Guidance on the EU Menu Methodology”, guidelines designed to refine the methods and protocols described previously, and to indicate criteria for the collection of high-quality dietary information. Despite this, some authors have refuted the use of M-BMs because of the non-quantifiable nature of the error of self-reported data, and the fact that they are derived from non-empirical phenomena which are prone to omissions, false memories, intentional misreporting and misestimating [23,24,43]. However, in the ANIBES study, we used new tools like tablets and digital cameras as more accurate and objective measures of estimating food and nutrient intakes. Likewise, two pilot studies were performed before the beginning of the main study to optimise the procedures and minimise the limitations of M-BMs.

Previously, reported ANIBES results, addressed the reported intake of energy and the main macronutrients i.e. carbohydrates, lipids and proteins [30,31,32].

In the ANIBES study, the mean reported intake of calcium in all age groups was much lower than the national [35] and European (EFSA) [36] recommendations, even when the plausible reporters, whose reported intake was higher than the whole population, were taken separately. The calcium intake for the Spanish population in The Spanish National Survey of Dietary Intake, (Encuesta Nacional de Ingesta Dietética España, ENIDE) [44] for adults was around 900 mg/day, with very slight differences between sexes. In the present study, the reported intake of calcium for the whole population was 698 mg/day, but 862 mg/day for the plausible reporters; this last value is very similar to the one reported in the ENIDE study [44], although it used a different methodology. Previous studies have been performed in other European countries, indicating the consumption of calcium in the adult population. Germany [45], the Netherlands [46] and Finland [47] have reported mean intakes over 1000 mg/day; France [48], Italy [49] and Portugal [48] around 900 mg/day; and Greece [48] and the UK [50] around 850 mg/day. In general, all these countries have a low percentage of the population not meeting the recommended daily intakes [51].

A recent review regarding low nutrient intake in nine European countries (Belgium, Denmark, France, Germany, the Netherlands, Poland, Serbia, Spain, and the UK) [52] indicates that the intake of calcium in children ranges from 563 mg/day to 1106 mg/day; in adolescents from 651 mg/day to 1487 mg/day; in adults from 512 mg/day to 1329 mg/day and in older adults from 529 mg/day to 1031 mg/day. Comparing these results with those obtained in the ANIBES study, show that calcium children’s reported intake was around the mean intake of the European countries included in the review, whereas adolescents, adults, and older adults were closer to the lower intakes. When only the plausible reporters were considered, adolescents, adults, and older adults’ calcium reported intakes were higher, closer to the European countries mean intakes according to their age and sex groups, and, in some cases even superior to those values.

As expected, milk and dairy products were the main source of calcium for the ANIBES population, as was observed in the ENIDE study [44]. However, the other foods’ groups contributed to the reported intake of calcium were in different order and proportions. It is interesting to observe that contrary to many other countries in Europe [53], fish represents a good source of calcium for the Spanish population, specifically in the adult and elderly groups. Conversely, meat and meat products contribute only to a low percentage (3% in both Spanish studies) of the global intake of calcium.

In all studied groups, the reported intake of phosphorus met almost the totality of the Spanish [35] recommendations as well as the European [36]. The plausible reporter’s reported intake was adequate, and 100% of the population met the recommendations. Phosphorus is neither a shortfall nor an over-consumed mineral in this studied population. This mineral can be considered as sufficient in the majority of countries and, in some age groups even excessive [48].

In contrast to the ENIDE study [44], where fish was the main dietary source of phosphorus, in ANIBES, milk and milk products ranked first. In both cases, meat and meat products were in second place with similar percentages. However, the other food groups were positioned in a different order.

The body regulation of phosphorus is close related to calcium. The recommended intake ratios for these minerals range from 1:1 to 1.5:1. As the consumption of calcium uses to be lower than the recommended daily intake and the phosphorus higher, this ratio uses to be lower than the recommended one. Consequently, there is more predisposition to bone resorption, low peak bone mass and increased bone fragility [54]. In the ANIBES study, the ratio for the whole population was 0.60 (0.61 for women and 0.58 for men), the same ratio as for the plausible reporters separately, very low compared with the recommendations.

The reported intakes of magnesium of the studied population were much lower than the Spanish [35] and European [36] recommendations, in both the whole population and the plausible reporters. The mean of the observed intake of magnesium in the ENIDE study [44] was around 350 mg/day; adult intake was 379 mg/day for females and 409 mg/day for males. According to that survey, only 30% had an inadequate intake of this nutrient. The data from the ANIBES study were much lower, as the mean reported intake for the whole population was 222 mg/day and for the plausible reporter’s group alone 273 mg/day. This disparity might be because each study identified different patterns of consumption for food with high content of magnesium, namely cereals, legumes and nuts.

The results of surveys from most of the European countries indicated that the intake of magnesium was below the respective recommendations and that the inadequacy was higher in women than in men in some age groups, but not in all [48]. Specifically, data from the national surveys indicated that Finnish [55] and Swedish [56] adult intake of magnesium was over 330 mg/day for females and over 400 mg/day for males. In the Netherlands [57], Italy [58] and Ireland [59], the intake for women was between 275 mg/day and 311 mg/day and for males over 340 mg/day and under 400 mg/day; and, in France [60] and the UK [61], the intakes were around 250 mg/day for females and less than 325 mg/day for males. All these intakes are higher than the intakes reported for the whole population in the ANIBES; however, when we take only the plausible reporters, the intakes are similar to the data reported by France and the UK. In their review of the EU countries, Mesnkink et al. [52] indicated that the magnesium intake in children ranged from 185 mg/day to 290 mg/day, in adolescents from 190 mg/day to 531 mg/day, in adults from 209 mg/day to 522 mg/day, and in older adults from 227 mg/day to 421 mg/day. When they excluded the under-reporters, the mean intake increased from 5% to 28% in the different age and sex groups. The ANIBES reported intake of magnesium for children was around the mean of the mentioned range, but for the other age groups, it was close to the lower reported values. However, when we took only the plausible reporters, the reported intakes increased in 10%, 12%, 24% and 38% in children, adolescents, adults, and older adults, respectively.

The main food source for magnesium reported intake in ANIBES was the group of cereals and grains, whereas in ENIDE it was the group of pulses and nuts. The remainder of the main groups that completed the list in ANIBES included milk and dairy products, meat and meat products and vegetables. However, in ENIDE [44] the main food group that followed pulses and nuts was fish.

Spanish DRV [35] for vitamin D are set for those individuals that have zero or very low endogenous synthesis of this micronutrient. Considering these recommendations, as well as those from EFSA [36], the reported intake of vitamin D was by far lower than the daily recommendations in both the whole population and the plausible reporters alone. In the ENIDE study [44], the observed intake of this vitamin was also very low, 3.65 µg/day for women and 4.28 µg/day for men. In fact, in ANIBES the reported intake was higher than in ENIDE for the whole population as well as for plausible reporters. Although the percentages of the population at risk of disparity between reported intake and the level needed for adequacy of Vitamin D in both Spanish studies were high and very similar. The same situation has been reported in most European countries. Norway is one of the countries that are above the recommendation; this is because it is mandatory, as it is in all Nordic countries, to fortify milk products and margarine, because of the relatively low sun exposure during winter months, and the potentially low sun exposure through a lifestyle that is dominated by indoor activities [52]. In detail, the intakes of this vitamin in some European countries are: Norway over 10 µg/day [52]; Finland [47] and Sweden [62] around 6 µg/day; Ireland [63] and Portugal [51] around 3.6 µg/day; and Italy [49], the Netherlands [46] and the UK [50] around 3.1 µg/day. In general, the prevalence of vitamin D inadequacy for these countries was 40%. The ranges for the intake of vitamin D according to national surveys in nine European countries were for children 1.3 µg/day to 3.5 µg/day, adolescents 1.5 µg/day to 4.8 µg/day, adults 1.7 µg/day to 6 µg/day, and for older adults 0.7 µg/day to 5.2 µg/day. Comparing with data of the Menskin et al. review [52], the ANIBES mean reported intakes of Vitamin D for all age groups were higher than European countries reported values. Although vitamin D3 is synthesised in the skin by the action of ultraviolet light, data from across the world indicate that hypovitaminosis D is widespread, even in those countries considered sunny, and it is currently a global major public health problem [64]. Deficiency of vitamin D has been reported in some selected Spanish populations. This fact is perceived to be due to the use of sunscreen lotions and sedentary behaviour, which as previously mentioned, avoids frequent sun exposure [65].

The ANIBES study has several strengths, which include the careful design, protocol, and methodology used, conducted among a random representative sample of the Spanish population aged 9–75 years. It is the first Spanish study at national level that analysed the data for the whole population and the plausible reporters. One limitation of this study is its cross-sectional design, which provides evidence for associations but not causal relationships [41].

## 5. Conclusions

The reported intake of calcium and magnesium are low in the ANIBES population. In the whole studied group, 76% and 66%, and 79% and 72% of the population had reported intakes below the 80% of the national and European recommended daily intakes respectively; even when the plausible reporters, whose reported intakes were higher than the whole population, were analysed separately. Vitamin D reported intake is inadequate when compared to the level needed for adequacy, 94% and 93% of the population had reported intakes below 80% of the daily recommendations according to the national and European references. The main food sources of calcium and phosphorus were milk and dairy products, for magnesium were cereals and grains and for vitamin D it was fish. The results indicate that there is an important percentage of the Spanish ANIBES population not meeting the current recommended intakes for calcium, magnesium and vitamin D, even when considering only the plausible reporters.

## Figures and Tables

**Table 1:** Calculated misreporting cut-off at group and individual levels for the ANIBES study.

	Misreporting Cut-Off			
	Group Level		Individual Level	
PAL	Lower	Upper	Lower	Upper
***Children and adoles* cents**				
1.6	1.55	1.66	0.93	2.76
1.8	1.73	1.86	1.04	3.10
2.0	1.93	2.07	1.16	3.45
***Adults and elderly***				
1.4	1.38	1.42	0.77	2.53
1.6	1.58	1.62	0.88	2.89
1.8	1.77	2.83	1.00	3.25

PAL: Physical activity level. The PAL is established for children and adolescents; and adults and elderly in three levels, low 1.6 and 1.4; moderate 1.8 and 1.6; and vigorous 2.0 and 1.8, respectively.

**Table 2:** Daily calcium, phosphorus, magnesium and vitamin D reported intake by sex and age group in the ANIBES Study population.

	Total			Children 9–12 Years			Adolescents 13–17 Years			Adults 18–64 Years			Elderly 65–75 Years		
	*n*	Mean ± SEM	Median (Range)	*n*	Mean ± SEM	Median (Range)	*n*	Mean ± SEM	Median (Range)	*n*	Mean ± SEM	Median (Range)	*n*	Mean ± SEM	Median (Range)
**CALCIUM (mg/day)**															
**Total**	2009	698 ± 7	664 (71–2551)	213	826 ± 17 ^a^	822 (302–1520)	211	817 ± 23 ^a^	774 (155–1960)	1655	689 ± 7 ^b^	659 (71–2551)	206	645 ± 19 ^b^	622 (157–2206)
*Men*	1013	726 ± 10 *	681 (71–2551)	126	872 ± 22 *	856 (302–1520)	137	875 ± 31 *	831 (174–1960)	798	711 ± 11 *	668 (71–2551)	99	662 ± 31	642 (223–2206)
*Women*	996	670 ± 8	650 (118–2399)	87	759 ± 26	763 (305–1500)	74	708 ± 29	667 (155–1276)	857	668 ± 9	650 (118–2399)	107	629 ± 21	613 (157–1344)
**PHOSPHORUS (mg/day)**															
**Total**	2009	1176 ± 8	1134 (331–4429)	213	1285 ± 22 ^a^	1286 (433–2086)	211	1261 ± 24 ^a^	1240 (414–2268)	1655	1175 ± 9 ^b^	1128 (331–4429)	206	1097 ± 23 ^c^	1058 (453–2921)
*Men*	1013	1246 ± 12 *	1200 (433–4429)	126	1340 ± 27 *	1362 (433–2086)	137	1323 ± 31 *	1310 (441–2268)	798	1247 ± 14 *	1197 (474–4429)	99	1177 ± 38 *	1117 (463–2921)
*Women*	996	1104 ± 9	1080 (331–2396)	87	1206 ± 35	1184 (646–1972)	74	1145 ± 35	1140 (414–1953)	857	1108 ± 10	1082 (331–2396)	107	1023 ± 25	1014 (453–1919)
**MAGNESIUM (mg/day)**															
**Total**	2009	222 ± 2	213 (73–782)	213	220 ± 4	221 (75–471)	211	216 ± 4	214 (80–410)	1655	223 ± 2	213 (73–782)	206	226 ± 6	210 (103–736)
*Men*	1013	233 ± 2 *	224 (80–782)	126	224 ± 5	222 (75–427)	137	224 ± 5 *	224 (80–410)	798	236 ± 3 *	225 (80–782)	99	246 ± 11 *	222 (110–736)
*Women*	996	210 ± 2	204 (73–592)	87	214 ± 7	213 (124–471)	74	200 ± 7	195 (93–387)	857	211 ± 2	204 (73–592)	107	207 ± 6	201 (103–430)
**VITAMIN D (µg/day)**															
**Total**	2009	4.4 ± 0.1	2.6 (0.0–74.2)	213	2.8 ± 0.2 ^a^	1.7 (0.1–13.5)	211	3.7 ± 0.4 ^a,b^	1.8 (0.0–73.9)	1655	4.5 ± 0.1 ^b^	2.9 (0.0–74.2)	206	4.4 ± 0.4 ^b^	2.8 (0.0–34.2)
*Men*	1013	4.4 ± 0.2	2.6 (0.0–74.2)	126	2.6 ± 0.2	1.7 (0.1–12.2)	137	4.0 ± 0.6	2.0 (0.0–73.9)	798	4.7 ± 0.2	2.8 (0.0–74.2)	99	4.5 ± 0.5	3.2 (0.0–30.7)
*Women*	996	4.3 ± 0.2	2.7 (0.0–47.2)	87	3.0 ± 0.3	1.8 (0.2–13.5)	74	3.1 ± 0.5	1.2 (0.1–29.4)	857	4.4 ± 0.2	2.9 (0.0–47.2)	107	4.3 ± 0.5	2.6 (0.1–34.6)

Results are expressed as the mean ± standard error of the mean (SEM) and median with range (in brackets); (*) *t*-tests or Mann–Whitney U test were used to evaluate differences by sex within the whole population and within each age group. ANOVA or Kruskal–Wallis analysis were used to calculate differences among age groups (mean values within the same row with unlike superscript letters were significantly different). *p* < 0.05 was considered statistically significant.

**Table 3:** Daily calcium, phosphorus, magnesium and vitamin D reported intake by plausible reporters, non-plausible reporters and age group in the ANIBES Study population.

	*Total*			*Children 9–12 Years*			*Adolescents 13–17 Years*			*Adults 18–64 Years*			*Elderly 65–75 Years*		
	*n*	Mean ± SEM	Median (Range)	*n*	Mean ± SEM	Median (Range)	*n*	Mean ± SEM	Median (Range)	*n*	Mean ± SEM	Median (Range)	*n*	Mean ± SEM	Median (Range)
**CALCIUM (mg/day)**															
**Total**	2009	698 ± 7	664 (71–2551)	213	826 ± 17	822 (302–1520)	211	817 ± 23	774 (155–1960)	1655	689 ± 7	659 (71–2551)	206	645 ± 19	622 (157–2206)
*Plausible reporters*	543	862 ± 14	824 (265–2399)	120	893 ± 22	876 (337–1520)	76	978 ± 39	959 (324–1960)	433	853 ± 16	790 (265–2399)	45	807 ± 47	795 (300–1943)
*Men*	232	934 ± 23	871 (300–2109)	68	954 ± 29	940 (337–1520)	48	1049 ± 55	991 (324–1960)	158	938 ± 29	851 (71–2551)	24	857 ± 71	821 (300–1943)
*Women*	311	807 ± 16	778 (265–2399)	52	812 ± 32	821 (354–1493)	28	855 ± 43	834 (527–1276)	275	804 ± 17	761 (265–2399)	21	750 ± 59	705 (363–1344)
*Non–Plausible reporters*	1466	637 ± 7	613 (71–2551)	93	739 ± 25	770 (302–1500)	135	726 ± 26	675 (155–1855)	1222	631 ± 7	605 (71–2551)	161	600 ± 18	572 (157–2206)
*Men*	781	664 ± 10	631 (71–2551)	58	775 ± 30	776 (302–1342)	89	782 ± 35	725 (174–1855)	640	655 ± 11	625 (71–2551)	75	600 ± 31	553 (223–2206)
*Women*	685	607 ± 9	585 (118–1684)	35	681 ± 41	637 (305–1500)	46	619 ± 32	627 (155–1088)	582	604 ± 9	579 (118–1684)	86	600 ± 21	590 (157–1052)
**PHOSPHORUS (mg/day)**															
**Total**	2009	1176 ± 8	1134 (331–4429)	213	1285 ± 22	1286 (433–2086)	211	1261 ± 24	1240 (414–2268)	1655	1175 ± 9	1128 (331–4429)	206	1097 ± 23	1058 (453–2921)
*Plausible reporters*	543	1434 ± 14	1401 (708–3300)	120	1408 ± 24	1395 (795–2086)	76	1502 ± 33	1502 (883–2268)	433	1433 ± 17	1396 (708–3300)	45	1410 ± 51	1392 (885–2415)
*Men*	232	1587 ± 23	1532 (901–3300)	68	1456 ± 29	1447 (949–2086)	48	1583 ± 41	1548 (984–2268)	158	1638 ± 30	1600 (901–3300)	24	1537 ± 72	1469 (928–2415)
*Women*	311	1321 ± 16	1304 (708–2396)	52	1344 ± 39	1317 (795–1972)	28	1362 ± 48	1329 (883–1953)	275	1315 ± 17	1288 (708–2396)	21	1265 ± 57	1270 (885–1919)
*Non–Plausible reporters*	1466	1080 ± 8	1048 (331–4429)	93	1128 ± 32	1098 (433–1989)	135	1125 ± 26	1111 (414–2083)	1222	1084 ± 9	1047 (331–4429)	161	1009 ± 21	993 (453–2921)
*Men*	781	1145 ± 12	1114 (433–4429)	58	1203 ± 40	1189 (433–1989)	89	1183 ± 34	1162 (441–2083)	640	1151 ± 13	1119 (474–4429)	75	1061 ± 36	1034 (463–2921)
*Women*	685	1006 ± 10	991 (331–1920)	35	1002 ± 46	960 (646–1756)	46	1012 ± 36	1021 (414–1558)	582	1010 ± 11	987 (331–1929)	86	963 ± 24	982 (453–1749)
**MAGNESIUM (mg/day)**															
**Total**	2009	222 ± 2	213 (73–782)	213	220 ± 4	221 (75–471)	211	216 ± 4	214 (80–410)	1655	223 ± 2	213 (73–782)	206	226 ± 6	210 (103–736)
*Plausible reporters*	543	273 ± 3	261 (122–669)	120	242 ± 5	241 (142–427)	76	263 ± 6	255 (166–410)	433	278 ± 4	266 (122–669)	45	300 ± 16	26 (165–736)
*Men*	232	301 ± 5	287 (154–669)	68	248 ± 6	248 (146–427)	48	274 ± 8	275 (178–410)	158	319 ± 6	300 (188–669)	24	327 ± 27	266 (184–736)
*Women*	311	253 ± 4	244 (122–592)	52	233 ± 7	237 (142–389)	28	242 ± 9	235 (166–387)	275	254 ± 4	246 (122–592)	21	269 ± 15	253 (165–430)
*Non–Plausible reporters*	1466	203 ± 2	197 (73–782)	93	192 ± 6	182 (75–471)	135	189 ± 4	188 (80–298)	1222	204 ± 2	197 (73–782)	161	205 ± 5	200 (101–641)
*Men*	781	214 ± 2	207 (80–782)	58	196 ± 7	189 (75–350)	89	197 ± 5	192 (80–298)	640	215 ± 3	207 (80–782)	75	220 ± 9	210 (110–641)
*Women*	685	191 ± 2	188 (73–591)	35	185 ± 11	179 (124–471)	46	175 ± 6	172 (93–253)	582	191 ± 2	187 (73–591)	86	192 ± 5	188 (103–327)
**VITAMIN D (µg/day)**															
**Total**	2009	4.4 ± 0.1	2.6 (0.0–74.2)	213	2.8 ± 0.2	1.7 (0.1–13.5)	211	3.7 ± 0.4	1.8 (0.0–73.7)	1655	4.5 ± 0.1	2.9 (0.0–74.2)	206	4.4 ± 0.4	2.8 (0.0–34.6)
*Plausible reporters*	543	5.5 ± 0.3	3.6 (0.1–47.2)	120	3.2 ± 0.3	2.0 (0.2–12.8)	76	3.8 ± 0.5	2.0 (0.1–20.9)	433	5.8 ± 0.3	4.1 (0.1–47.2)	45	6.7 ± 0.9	5.2 (0.4–30–7)
*Men*	232	5.5 ± 0.4	3.9 (0.1–38.3)	68	3.0 ± 0.3	1.9 (0.2–12.2)	48	4.0 ± 0.6	2.0 (0.1–20.9)	158	5.9 ± 0.5	4.4 (0.1–38.3)	24	8.0 ± 1.3	6.3 (1.0–30.7)
*Women*	311	5.5 ± 0.4	3.5 (0.1–47.2)	52	3.3 ± 0.4	2.5 (0.2–12.8)	28	3.3 ± 0.8	1.5 (0.4–18.2)	275	5.7 ± 0.4	3.9 (0.1–47.2)	21	5.2 ± 1.2	3.3 (0.4–20.9)
*Non–Plausible reporters*	1466	4.0 ± 0.1	2.3 (0.0–74.2)	93	2.3 ± 0.2	1.3 (0.1–13.5)	135	3.7 ± 0.6	1.8 (0.0–73.9)	1222	4.1 ± 0.1	2.5 (0.0–74.2)	161	3.8 ± 0.4	2.2 (0.0–34.6)
*Men*	781	4.1 ± 0.2	2.3 (0.0–74.2)	58	2.2 ± 0.3	1.3 (0.1–9.2)	89	4.0 ± 0.9	1.9 (0.0–73.9)	640	4.3 ± 0.2	2.5 (0.0–74.2)	75	3.4 ± 0.4	2.2 (0.0–15.6)
*Women*	685	3.8 ± 0.2	2.3 (0.0–37.2)	35	2.5 ± 0.5	1.5 (0.2–13.5)	46	2.9 ± 0.7	1.0 (0.0–29.4)	582	3.7 ± 0.2	2.4 (0.0–37.2)	86	4.1 ± 0.6	2.2 (0.1–34.6)

Results are expressed as the mean ± standard error of the mean and median with range (in brackets). There were significant differences between plausible and non-plausible reporters for the whole population and within sexes into each age group (*p* < 0.05), except for vitamin D in the adolescents.

**Table 4:** Percentage of the population with disparity between reported intake and the level needed for adequacy for calcium, magnesium and vitamin D for the whole population and for the plausible reporters by age.

	*Total*		*Children 9–12 Years*		*Adolescents 13–17 Years*		*Adults 18–64 Years*		*Elderly 65–75 Years*	
	*Spain*	*EFSA*	*Spain*	*EFSA*	*Spain*	*EFSA*	*Spain*	*EFSA*	*Spain*	*EFSA*
**CALCIUM (%)**										
*Whole population*	76	66	62	38	78	65	74	66	90	73
*Men*	72	63	57	29	71	58	70	64	88	71
*Women*	80	69	68	53	91	77	78	68	92	75
*Plausible reporters*	56	44	48	23	65	45	55	47	67	49
*Men*	49	37	44	12	54	35	45	40	63	46
*Women*	61	50	52	38	79	61	60	51	71	52
**MAGNESIUM (%)**										
*Whole population*	79	72	65	48	90	57	78	73	79	79
*Men*	78	74	69	53	91	60	76	76	81	81
*Women*	80	70	59	40	89	51	80	71	77	77
*Plausible reporters*	53	40	49	29	72	26	51	41	44	44
*Men*	45	38	57	34	73	29	35	35	54	54
*Women*	58	42	38	23	71	21	60	45	33	33
**VITAMIN D (%)**										
*Whole population*	94	93	99	99	95	95	93	93	97	94
*Men*	93	93	99	99	94	94	92	92	99	93
*Women*	94	94	98	98	97	97	94	94	95	94
*Plausible reporters*	90	89	98	98	93	93	89	88	94	84
*Men*	91	89	99	99	92	92	88	88	96	79
*Women*	89	89	98	98	96	96	89	89	90	90

Results are expressed in percentage. Recommended daily intakes for Spain [35] and Europe [36]. Adequacy was calculated comparing with 80% of the Spanish DRV and EFSA PRI or AI.

## Supplementary Materials

The following are available online at http://www.mdpi.com/2072-6643/9/2/168/s1.
