# Supplementary material for: Reported Dietary Intake, Disparity between the Reported Consumption and the Level Needed for Adequacy and Food Sources of Calcium, Phosphorus, Magnesium and Vitamin D in the Spanish Population: Findings from the ANIBES Study
Source: Nutrients. 2017 Feb 21;9(2):168. doi: 10.3390/nu9020168 (PMC5331599; doi:10.3390/nu9020168)
Supplement: Supplementary file 1 [file nutrients-09-00168-s001.docx]

Supplementary Materials: Reported Dietary Intake, Disparity between the Reported Consumption and the Level Needed for Adequacy and Food Sources of Calcium, Phosphorus, Magnesium and Vitamin D in the Spanish Population: Findings from the ANIBES Study ^†^

Josune Olza, Javier Aranceta-Bartrina, Marcela González-Gross, Rosa M. Ortega, Lluis Serra-Majem, Gregorio Varela-Moreiras and Ángel Gil

**Table S1. Daily calcium intake and distribution by sex and age group in the ANIBES Study population.**

| **CALCIUM (mg/d)** | **n** | **Mean** | **SD** | **SEM** | **P5** | **P25** | **P50 Median** | **P75** | **P95** | **Minimum** | **Maximum** |
| --- | --- | --- | --- | --- | --- | --- | --- | --- | --- | --- | --- |
| **Total** | **2009** | **698** | **295** | **7** | **298** | **492** | **664** | **854** | **1220** | **71** | **2551** |
| Men | 1013 | 726 | 322 | 10 | 302 | 497 | 681 | 897 | 1324 | 71 | 2551 |
| Women | 996 | 670 | 263 | 8 | 295 | 491 | 650 | 820 | 1113 | 118 | 2399 |
| **Children 9–12 years** | **213** | **826** | **254** | **17** | **397** | **663** | **822** | **973** | **1317** | **302** | **1520** |
| Men | 126 | 872 | 251 | 22 | 440 | 698 | 856 | 1012 | 1340 | 302 | 1520 |
| Women | 87 | 759 | 244 | 26 | 369 | 590 | 763 | 911 | 1144 | 305 | 1500 |
| **Adolescents 13–17 years** | **211** | **817** | **340** | **23** | **308** | **567** | **774** | **983** | **1506** | **155** | **1960** |
| Men | 137 | 875 | 368 | 31 | 403 | 607 | 831 | 1090 | 1576 | 174 | 1960 |
| Women | 74 | 708 | 248 | 29 | 278 | 546 | 667 | 869 | 1125 | 155 | 1276 |
| **Adults 18–64 years** | **1655** | **689** | **294** | **7** | **290** | **484** | **659** | **845** | **1209** | **71** | **2551** |
| Men | 798 | 711 | 320 | 11 | 284 | 483 | 668 | 872 | 1317 | 71 | 2551 |
| Women | 857 | 668 | 266 | 9 | 295 | 487 | 650 | 812 | 1113 | 118 | 2399 |
| **Elderly 65–75 years** | **206** | **645** | **266** | **19** | **283** | **461** | **622** | **804** | **1052** | **157** | **2206** |
| Men | 99 | 662 | 310 | 31 | 304 | 444 | 642 | 819 | 1181 | 223 | 2206 |
| Women | 107 | 629 | 219 | 21 | 270 | 486 | 613 | 763 | 1019 | 157 | 1344 |

**Table S2. Daily phosphorus intake and distribution by sex and age group in the ANIBES Study population.**

| **PHOSPHORUS (mg/d)** | **n** | **Mean** | **SD** | **SEM** | **P5** | **P25** | **P50 Median** | **P75** | **P95** | **Minimum** | **Maximum** |
| --- | --- | --- | --- | --- | --- | --- | --- | --- | --- | --- | --- |
| **Total** | **2009** | **1176** | **351** | **8** | **678** | **938** | **1134** | **1372** | **1790** | **331** | **4429** |
| Men | 1013 | 1246 | 385 | 12 | 698 | 986 | 1200 | 1443 | 1906 | 433 | 4429 |
| Women | 996 | 1104 | 298 | 9 | 665 | 897 | 1080 | 1290 | 1641 | 331 | 2396 |
| **Children 9–12 years** | **213** | **1285** | **316** | **22** | **767** | **1075** | **1286** | **1487** | **1809** | **433** | **2086** |
| Men | 126 | 1340 | 300 | 27 | 869 | 1148 | 1362 | 1507 | 1845 | 433 | 2086 |
| Women | 87 | 1206 | 323 | 35 | 738 | 946 | 1184 | 1415 | 1773 | 646 | 1972 |
| **Adolescents 13–17 years** | **211** | **1261** | **350** | **24** | **738** | **1007** | **1240** | **1495** | **1898** | **414** | **2268** |
| Men | 137 | 1323 | 360 | 31 | 752 | 1052 | 1310 | 1550 | 2028 | 441 | 2268 |
| Women | 74 | 1145 | 301 | 35 | 676 | 897 | 1140 | 1336 | 1643 | 414 | 1953 |
| **Adults 18–64 years** | **1655** | **1175** | **354** | **9** | **679** | **935** | **1128** | **1372** | **1798** | **331** | **4429** |
| Men | 798 | 1247 | 392 | 14 | 699 | 984 | 1197 | 1443 | 1912 | 474 | 4429 |
| Women | 857 | 1108 | 300 | 10 | 671 | 902 | 1082 | 1295 | 1644 | 331 | 2396 |
| **Elderly 65–75 years** | **206** | **1097** | **332** | **23** | **650** | **888** | **1058** | **1256** | **1641** | **453** | **2921** |
| Men | 99 | 1177 | 380 | 38 | 681 | 947 | 1117 | 1358 | 1925 | 463 | 2921 |
| Women | 107 | 1023 | 261 | 25 | 567 | 865 | 1014 | 1190 | 1399 | 453 | 1919 |

**Table S3. Daily magnesium intake and distribution by sex and age group in the ANIBES Study population.**

| **MAGNESIUM (mg/d)** | **n** | **Mean** | **SD** | **SEM** | **P5** | **P25** | **P50 Median** | **P75** | **P95** | **Minimum** | **Maximum** |
| --- | --- | --- | --- | --- | --- | --- | --- | --- | --- | --- | --- |
| **Total** | **2009** | **222** | **72** | **2** | **127** | **174** | **213** | **257** | **347** | **73** | **782** |
| Men | 1013 | 233 | 78 | 2 | 129 | 181 | 224 | 273 | 367 | 80 | 782 |
| Women | 996 | 210 | 63 | 2 | 125 | 166 | 204 | 245 | 323 | 73 | 592 |
| **Children 9–12 years** | **213** | **220** | **59** | **4** | **130** | **179** | **221** | **256** | **318** | **75** | **471** |
| Men | 126 | 224 | 58 | 5 | 129 | 182 | 222 | 263 | 318 | 75 | 427 |
| Women | 87 | 214 | 61 | 7 | 131 | 166 | 213 | 247 | 306 | 124 | 471 |
| **Adolescents 13–17 years** | **211** | **216** | **61** | **4** | **123** | **169** | **214** | **251** | **324** | **80** | **410** |
| Men | 137 | 224 | 63 | 5 | 133 | 178 | 224 | 260 | 330 | 80 | 410 |
| Women | 74 | 200 | 56 | 7 | 120 | 160 | 195 | 236 | 288 | 93 | 387 |
| **Adults 18–64 years** | **1655** | **223** | **73** | **2** | **125** | **174** | **213** | **259** | **350** | **73** | **782** |
| Men | 798 | 236 | 80 | 3 | 127 | 181 | 225 | 277 | 372 | 80 | 782 |
| Women | 857 | 211 | 64 | 2 | 123 | 166 | 204 | 247 | 323 | 73 | 592 |
| **Elderly 65–75 years** | **206** | **226** | **87** | **6** | **135** | **174** | **210** | **248** | **400** | **103** | **736** |
| Men | 99 | 246 | 106 | 11 | 137 | 192 | 222 | 260 | 541 | 110 | 736 |
| Women | 107 | 207 | 58 | 6 | 131 | 167 | 201 | 239 | 318 | 103 | 430 |

**Table S4. Daily vitamin D intake and distribution by sex and age group in the ANIBES Study population.**

| **VITAMIN D (µg/d)** | **n** | **Mean** | **SD** | **SEM** | **P5** | **P25** | **P50 Median** | **P75** | **P95** | **Minimum** | **Maximum** |
| --- | --- | --- | --- | --- | --- | --- | --- | --- | --- | --- | --- |
| **Total** | **2009** | **4.4** | **5.2** | **0.1** | **0.2** | **0.9** | **2.6** | **6.0** | **13.4** | **0.0** | **74.2** |
| Men | 1013 | 4.4 | 5.2 | 0.2 | 0.2 | 1.0 | 2.6 | 6.1 | 13.5 | 0.0 | 74.2 |
| Women | 996 | 4.3 | 5.2 | 0.2 | 0.2 | 0.8 | 2.7 | 5.9 | 13.0 | 0.0 | 47.2 |
| **Children 9–12 years** | **213** | **2.8** | **2.7** | **0.2** | **0.3** | **0.9** | **1.7** | **3.7** | **9.3** | **0.1** | **13.5** |
| Men | 126 | 2.6 | 2.5 | 0.2 | 0.4 | 0.9 | 1.7 | 3.5 | 7.1 | 0.1 | 12.2 |
| Women | 87 | 3.0 | 3.0 | 0.3 | 0.2 | 0.8 | 1.7 | 4.4 | 10.4 | 0.2 | 13.5 |
| **Adolescents 13–17 years** | **211** | **3.7** | **6.4** | **0.4** | **0.3** | **0.7** | **1.8** | **4.6** | **11.9** | **0.0** | **73.9** |
| Men | 137 | 4.0 | 7.2 | 0.6 | 0.3 | 1.0 | 2.0 | 5.1 | 12.3 | 0.0 | 73.9 |
| Women | 74 | 3.1 | 4.6 | 0.5 | 0.2 | 0.7 | 1.2 | 3.9 | 11.3 | 0.1 | 29.4 |
| **Adults 18–64 years** | **1655** | **4.5** | **5.3** | **0.1** | **0.2** | **0.9** | **2.9** | **6.1** | **13.6** | **0.0** | **74.2** |
| Men | 798 | 4.7 | 5.5 | 0.2 | 0.2 | 1.0 | 2.8 | 6.5 | 14.0 | 0.0 | 74.2 |
| Women | 857 | 4.4 | 5.1 | 0.2 | 0.2 | 0.9 | 2.9 | 6.0 | 13.0 | 0.0 | 47.2 |
| **Elderly 65–75 years** | **206** | **4.4** | **5.2** | **0.4** | **0.2** | **0.8** | **2.8** | **6.2** | **14.2** | **0.0** | **34.6** |
| Men | 99 | 4.5 | 4.7 | 0.5 | 0.1 | 1.0 | 3.2 | 6.3 | 14.3 | 0.0 | 30.7 |
| Women | 107 | 4.3 | 5.5 | 0.5 | 0.3 | 0.8 | 2.6 | 6.2 | 14.2 | 0.1 | 34.6 |

**Table S5. Dietary sources of calcium (%) from food groups/subgroups by sex and age groups in the ANIBES Spanish population.**

| **CALCIUM** | **Total 9–75** | **Children 9–12** | **Adolescents 13–17** | **Adults 18–64** | **Elderly 65–75** |
| --- | --- | --- | --- | --- | --- |
| **(%)** | **2009** | **213** | **211** | **1655** | **206** |
| **Alcoholic beverages** | **1.69** | **-** | **0.02** | **1.91** | **1.80** |
| ***High alcohol content beverages*** | - | - | - | - | - |
| ***Low alcohol content beverages*** | 1.69 | - | 0.02 | 1.91 | 1.80 |
| **Appetizers** | **0.44** | **0.39** | **0.39** | **0.47** | **0.27** |
| **Cereals/Grains** | **11.19** | **13.65** | **15.21** | **11.03** | **8.83** |
| ***Grains and flours*** | 0.62 | 0.41 | 0.58 | 0.64 | 0.55 |
| ***Breakfast cereals and cereal bars*** | 0.74 | 2.11 | 2.72 | 0.56 | 0.09 |
| ***Bread*** | 5.26 | 5.56 | 6.20 | 5.33 | 4.16 |
| *White bread* | 4.58 | 5.34 | 5.85 | 4.60 | 3.43 |
| *Brown bread* | 0.48 | 0.14 | 0.19 | 0.52 | 0.60 |
| *Others breads* | 0.20 | 0.08 | 0.16 | 0.21 | 0.12 |
| ***Pasta*** | 0.87 | 0.74 | 1.10 | 0.91 | 0.50 |
| ***Bakery and pastry*** | 3.69 | 4.82 | 4.61 | 3.59 | 3.53 |
| **Eggs** | **2.78** | **2.04** | **2.80** | **2.79** | **3.20** |
| **Fish** | **4.23** | **2.33** | **2.44** | **4.30** | **5.62** |
| ***White fish*** | 0.90 | 0.83 | 0.49 | 0.84 | 1.72 |
| ***Oily fish*** | 0.52 | 0.24 | 0.28 | 0.52 | 0.72 |
| ***Shellfish*** | 1.81 | 1.01 | 1.10 | 1.89 | 2.03 |
| ***Canned fish*** | 1.00 | 0.25 | 0.56 | 1.05 | 1.15 |
| **Fruits** | **3.63** | **1.85** | **1.59** | **3.57** | **6.31** |
| **Meat and meat products** | **3.43** | **2.92** | **3.63** | **3.52** | **2.63** |
| ***Meat*** | 2.30 | 1.80 | 2.39 | 2.37 | 1.78 |
| *Red meat* | 0.62 | 0.58 | 0.57 | 0.63 | 0.51 |
| *White meat* | 0.51 | 0.29 | 0.51 | 0.54 | 0.38 |
| *Poultry* | 1.17 | 0.92 | 1.31 | 1.19 | 0.89 |
| ***Viscera and offal*** | 0.03 | - | 0.01 | 0.03 | 0.04 |
| ***Sausages and other meat products*** | 1.11 | 1.12 | 1.23 | 1.12 | 0.81 |
| **Milk and dairy products** | **53.04** | **60.31** | **54.44** | **52.52** | **53.57** |
| ***Milk*** | 28.66 | 33.05 | 30.06 | 27.86 | 32.28 |
| *Whole milk* | 8.77 | 15.41 | 13.81 | 8.09 | 8.83 |
| *Semi skimmed milk* | 13.03 | 14.46 | 11.90 | 12.88 | 13.06 |
| *Skimmed milk* | 6.47 | 3.13 | 4.24 | 6.48 | 9.94 |
| *Other dairy* | 0.39 | 0.05 | 0.10 | 0.41 | 0.45 |
| ***Yogurt and fermented milk*** | 8.43 | 8.83 | 5.90 | 8.21 | 10.85 |
| *Skimmed Fermented milk* | 0.76 | 2.08 | 0.64 | 0.64 | 1.40 |
| *Whole fermented milk* | 0.56 | 0.11 | 0.17 | 0.58 | 0.67 |
| *Skimmed milk yogurts* | 2.13 | 0.32 | 0.48 | 2.19 | 2.90 |
| *Whole milk yogurt* | 4.97 | 6.32 | 4.60 | 4.80 | 5.88 |
| ***Cheese*** | 12.85 | 11.09 | 13.90 | 13.44 | 8.80 |
| ***Other dairy products*** | 3.10 | 7.34 | 4.58 | 3.00 | 1.65 |
| **Non-alcoholic beverages** | **2.51** | **1.56** | **2.15** | **2.64** | **2.11** |
| ***Water*** | - | - | - | - | - |
| ***Coffee and herbal teas*** | 0.68 | 0.02 | 0.07 | 0.73 | 0.96 |
| ***Sugared soft drinks*** | 0.49 | 0.30 | 0.75 | 0.51 | 0.12 |
| ***Unsweetened soft drinks*** | 0.21 | 0.05 | 0.11 | 0.25 | 0.08 |
| ***Sports drinks*** | 0.02 | 0.03 | 0.01 | 0.03 | 0.01 |
| ***Juices and nectars*** | 0.81 | 1.15 | 1.12 | 0.79 | 0.59 |
| ***Energy drinks*** | - | - | - | - | - |
| ***Other non-alcoholic beverages*** | 0.28 | 0.02 | 0.08 | 0.34 | 0.35 |
| **Oils and fats** | **0.08** | **0.06** | **0.08** | **0.08** | **0.10** |
| ***Olive oil*** | - | - | - | - | - |
| ***Other oils*** | - | - | - | - | - |
| ***Butter, margarine and shortening*** | 0.08 | 0.06 | 0.08 | 0.08 | 0.10 |
| **Pulses** | **2.12** | **1.45** | **1.80** | **2.12** | **2.48** |
| **Sauces and condiments** | **0.71** | **0.62** | **0.69** | **0.74** | **0.50** |
| **Sugars and sweets** | **0.87** | **1.93** | **1.77** | **0.81** | **0.45** |
| ***Sugar*** | 0.06 | 0.01 | 0.02 | 0.06 | 0.07 |
| ***Chocolate*** | 0.73 | 1.85 | 1.68 | 0.66 | 0.19 |
| ***Jams and other*** | 0.08 | 0.04 | 0.03 | 0.07 | 0.19 |
| ***Other sweets*** | 0.01 | 0.03 | 0.04 | 0.02 | - |
| **Supplements and meal replacements** | **0.22** | **-** | **-** | **0.24** | **0.34** |
| **Ready-to-eat-meals** | **5.11** | **6.45** | **8.56** | **5.06** | **2.27** |
| **Vegetables** | **7.93** | **4.43** | **4.42** | **8.20** | **9.53** |

**Table S6. Dietary sources of phosphorus (%) from food groups/subgroups by sex and age groups in the ANIBES Spanish population.**

| **PHOSPHORUS** | **Total 9–75** | **Children 9–12** | **Adolescents 13–17** | **Adults 18–64** | **Elderly 65–75** |
| --- | --- | --- | --- | --- | --- |
| **(%)** | **2009** | **213** | **211** | **1655** | **206** |
| **Alcoholic beverages** | **1.76** | **-** | **0.03** | **2.01** | **1.57** |
| ***High alcohol content beverages*** | 0.01 | - | - | 0.01 | 0.01 |
| ***Low alcohol content beverages*** | 1.75 | - | 0.03 | 2.00 | 1.56 |
| **Appetizers** | **0.38** | **0.67** | **0.59** | **0.38** | **0.09** |
| **Cereals/Grains** | **16.06** | **16.24** | **17.31** | **16.10** | **15.07** |
| ***Grains and flours*** | 2.64 | 2.13 | 2.61 | 2.68 | 2.37 |
| ***Breakfast cereals and cereal bars*** | 0.34 | 0.28 | 0.24 | 0.37 | 0.35 |
| ***Bread*** | 6.83 | 5.89 | 6.45 | 6.90 | 7.20 |
| *White bread* | 5.45 | 5.37 | 5.91 | 5.42 | 5.58 |
| *Brown bread* | 1.13 | 0.38 | 0.38 | 1.21 | 1.41 |
| *Others breads* | 0.25 | 0.14 | 0.16 | 0.26 | 0.21 |
| ***Pasta*** | 3.03 | 3.35 | 3.91 | 3.07 | 1.97 |
| ***Bakery and pastry*** | 3.21 | 4.58 | 4.10 | 3.08 | 3.17 |
| **Eggs** | **4.62** | **4.01** | **4.61** | **4.60** | **5.56** |
| **Fish** | **9.07** | **6.13** | **5.56** | **9.04** | **12.49** |
| ***White fish*** | 2.78 | 2.74 | 1.70 | 2.59 | 5.09 |
| ***Oily fish*** | 1.95 | 1.31 | 1.18 | 1.90 | 2.86 |
| ***Shellfish*** | 1.95 | 1.09 | 1.14 | 2.04 | 2.10 |
| ***Canned fish*** | 2.39 | 0.99 | 1.54 | 2.51 | 2.44 |
| **Fruits** | **2.98** | **1.77** | **1.53** | **2.94** | **5.08** |
| **Meat and meat products** | **19.56** | **18.15** | **20.38** | **19.86** | **16.54** |
| ***Meat*** | 13.72 | 11.71 | 13.90 | 13.99 | 11.95 |
| *Red meat* | 4.01 | 3.51 | 3.55 | 4.05 | 4.05 |
| *White meat* | 3.91 | 2.96 | 4.03 | 4.07 | 3.17 |
| *Poultry* | 5.80 | 5.24 | 6.32 | 5.87 | 4.73 |
| ***Viscera and offal*** | 0.33 | 0.04 | 0.09 | 0.34 | 0.48 |
| ***Sausages and other meat products*** | 5.51 | 6.40 | 6.40 | 5.53 | 4.10 |
| **Milk and dairy products** | **26.12** | **31.00** | **26.84** | **25.66** | **27.29** |
| ***Milk*** | 12.66 | 15.55 | 13.97 | 12.17 | 14.65 |
| *Whole milk* | 3.86 | 7.32 | 6.33 | 3.54 | 3.88 |
| *Semi skimmed milk* | 5.73 | 6.77 | 5.64 | 5.58 | 5.91 |
| *Skimmed milk* | 2.87 | 1.43 | 1.95 | 2.84 | 4.62 |
| *Other dairy* | 0.19 | 0.03 | 0.06 | 0.20 | 0.24 |
| ***Yogurt and fermented milk*** | 4.90 | 5.60 | 3.73 | 4.74 | 6.43 |
| *Skimmed Fermented milk* | 0.42 | 1.24 | 0.37 | 0.36 | 0.81 |
| *Whole fermented milk* | 0.28 | 0.06 | 0.09 | 0.28 | 0.34 |
| *Skimmed milk yogurts* | 1.07 | 0.17 | 0.25 | 1.09 | 1.52 |
| *Whole milk yogurt* | 3.13 | 4.13 | 3.02 | 3.01 | 3.77 |
| ***Cheese*** | 7.06 | 5.94 | 6.68 | 7.35 | 5.43 |
| ***Other dairy products*** | 1.49 | 3.92 | 2.47 | 1.41 | 0.77 |
| **Non-alcoholic beverages** | **2.60** | **1.52** | **2.18** | **2.75** | **2.09** |
| ***Water*** | - | - | - | - | - |
| ***Coffee and herbal teas*** | 0.47 | 0.01 | 0.05 | 0.49 | 0.77 |
| ***Sugared soft drinks*** | 0.80 | 0.54 | 1.12 | 0.83 | 0.21 |
| ***Unsweetened soft drinks*** | 0.39 | 0.10 | 0.20 | 0.45 | 0.16 |
| ***Sports drinks*** | - | 0.01 | - | - | - |
| ***Juices and nectars*** | 0.50 | 0.83 | 0.68 | 0.49 | 0.38 |
| ***Energy drinks*** | - | - | - | - | - |
| ***Other non-alcoholic beverages*** | 0.43 | 0.04 | 0.14 | 0.49 | 0.56 |
| **Oils and fats** | **0.06** | **0.05** | **0.05** | **0.06** | **0.07** |
| ***Olive oil*** | 0.02 | 0.02 | 0.02 | 0.02 | 0.03 |
| ***Other oils*** | - | - | - | - | - |
| ***Butter, margarine and shortening*** | 0.04 | 0.04 | 0.04 | 0.04 | 0.05 |
| **Pulses** | **3.26** | **2.65** | **2.86** | **3.21** | **4.13** |
| **Sauces and condiments** | **0.76** | **0.83** | **0.71** | **0.79** | **0.44** |
| **Sugars and sweets** | **2.82** | **7.48** | **6.47** | **2.56** | **0.96** |
| ***Sugar*** | 0.01 | - | 0.01 | 0.01 | 0.01 |
| ***Chocolate*** | 2.76 | 7.41 | 6.42 | 2.49 | 0.87 |
| ***Jams and other*** | 0.04 | 0.02 | 0.02 | 0.04 | 0.09 |
| ***Other sweets*** | 0.02 | 0.05 | 0.03 | 0.02 | - |
| **Supplements and meal replacements** | **0.06** | **-** | **-** | **0.07** | **-** |
| **Ready-to-eat-meals** | **4.07** | **5.76** | **6.91** | **4.00** | **1.52** |
| **Vegetables** | **5.82** | **3.75** | **3.94** | **5.96** | **7.11** |

**Table S7. Dietary sources of magnesium (%) from food groups/subgroups by sex and age groups in the ANIBES Spanish population.**

| **MAGNESIUM** | **Total 9–75** | **Children 9–12** | **Adolescents 13–17** | **Adults 18–64** | **Elderly 65–75** |
| --- | --- | --- | --- | --- | --- |
| **(%)** | **2009** | **213** | **211** | **1655** | **206** |
| **Alcoholic beverages** | **3.10** | **-** | **0.06** | **3.48** | **3.44** |
| ***High alcohol content beverages*** | - | - | - | - | - |
| ***Low alcohol content beverages*** | 3.10 | - | 0.06 | 3.48 | 3.44 |
| **Appetizers** | **0.90** | **1.53** | **1.42** | **0.91** | **0.21** |
| **Cereals/Grains** | **22.56** | **25.07** | **26.78** | **22.56** | **19.19** |
| ***Grains and flours*** | 2.79 | 2.48 | 2.84 | 2.82 | 2.42 |
| ***Breakfast cereals and cereal bars*** | 0.79 | 1.27 | 1.58 | 0.74 | 0.51 |
| ***Bread*** | 11.29 | 11.01 | 11.77 | 11.38 | 10.80 |
| *White bread* | 8.80 | 9.95 | 10.75 | 8.70 | 8.05 |
| *Brown bread* | 2.29 | 0.92 | 0.89 | 2.46 | 2.60 |
| *Others breads* | 0.21 | 0.13 | 0.13 | 0.22 | 0.15 |
| ***Pasta*** | 4.52 | 5.44 | 6.36 | 4.55 | 2.86 |
| ***Bakery and pastry*** | 3.18 | 4.88 | 4.23 | 3.08 | 2.60 |
| **Eggs** | **1.61** | **1.50** | **1.74** | **1.59** | **1.79** |
| **Fish** | 6.30 | 4.80 | 4.26 | 6.25 | 8.25 |
| ***White fish*** | 1.80 | 2.03 | 1.20 | 1.67 | 3.17 |
| ***Oily fish*** | 1.29 | 0.95 | 0.84 | 1.25 | 1.75 |
| ***Shellfish*** | 1.84 | 1.25 | 1.24 | 1.87 | 2.02 |
| ***Canned fish*** | 1.38 | 0.56 | 0.98 | 1.45 | 1.31 |
| **Fruits** | **8.61** | **6.36** | **5.09** | **8.43** | **13.38** |
| **Meat and meat products** | **12.46** | **12.35** | **14.06** | **12.61** | **9.74** |
| ***Meat*** | 9.24 | 8.59 | 10.23 | 9.38 | 7.42 |
| *Red meat* | 2.39 | 2.27 | 2.19 | 2.42 | 2.17 |
| *White meat* | 2.38 | 2.06 | 2.65 | 2.47 | 1.80 |
| *Poultry* | 4.47 | 4.26 | 5.39 | 4.50 | 3.45 |
| ***Viscera and offal*** | 0.13 | 0.03 | 0.02 | 0.14 | 0.17 |
| ***Sausages and other meat products*** | 3.09 | 3.73 | 3.81 | 3.09 | 2.15 |
| **Milk and dairy products** | **15.71** | **21.77** | **18.63** | **15.21** | **15.51** |
| ***Milk*** | 9.17 | 12.44 | 11.17 | 8.77 | 9.76 |
| *Whole milk* | 2.89 | 5.92 | 5.20 | 2.63 | 2.68 |
| *Semi skimmed milk* | 4.12 | 5.40 | 4.43 | 3.99 | 3.92 |
| *Skimmed milk* | 2.02 | 1.09 | 1.49 | 2.01 | 3.03 |
| *Other dairy* | 0.14 | 0.02 | 0.05 | 0.14 | 0.13 |
| ***Yogurt and fermented milk*** | 2.97 | 3.64 | 2.32 | 2.88 | 3.64 |
| *Skimmed Fermented milk* | 0.28 | 0.85 | 0.25 | 0.24 | 0.52 |
| *Whole fermented milk* | 0.18 | 0.05 | 0.06 | 0.19 | 0.20 |
| *Skimmed milk yogurts* | 0.74 | 0.11 | 0.20 | 0.76 | 0.98 |
| *Whole milk yogurt* | 1.76 | 2.62 | 1.81 | 1.69 | 1.95 |
| ***Cheese*** | 2.47 | 2.33 | 2.69 | 2.55 | 1.75 |
| ***Other dairy products*** | 1.10 | 3.37 | 2.45 | 1.01 | 0.35 |
| **Non-alcoholic beverages** | **5.32** | **3.33** | **3.58** | **5.53** | **5.85** |
| ***Water*** | - | - | - | - | - |
| ***Coffee and herbal teas*** | 2.61 | 0.09 | 0.31 | 2.77 | 3.78 |
| ***Sugared soft drinks*** | 0.31 | 0.22 | 0.45 | 0.32 | 0.07 |
| ***Unsweetened soft drinks*** | 0.15 | 0.04 | 0.08 | 0.17 | 0.05 |
| ***Sports drinks*** | 0.01 | 0.02 | - | 0.01 | - |
| ***Juices and nectars*** | 1.64 | 2.91 | 2.50 | 1.58 | 1.11 |
| ***Energy drinks*** | - | - | - | - | - |
| ***Other non-alcoholic beverages*** | 0.61 | 0.06 | 0.24 | 0.68 | 0.84 |
| **Oils and fats** | **0.03** | **0.03** | **0.03** | **0.03** | **0.03** |
| ***Olive oil*** | - | - | - | - | - |
| ***Other oils*** | - | - | - | - | - |
| ***Butter, margarine and shortening*** | 0.03 | 0.03 | 0.03 | 0.03 | 0.03 |
| **Pulses** | **5.41** | **4.93** | **5.04** | **5.32** | **6.42** |
| **Sauces and condiments** | **1.24** | **1.34** | **1.33** | **1.27** | **0.82** |
| **Sugars and sweets** | **1.21** | **2.80** | **2.34** | **1.14** | **0.50** |
| ***Sugar*** | 0.03 | 0.01 | 0.02 | 0.03 | 0.04 |
| ***Chocolate*** | 1.10 | 2.74 | 2.20 | 1.02 | 0.38 |
| ***Jams and other*** | 0.04 | 0.03 | 0.02 | 0.03 | 0.08 |
| ***Other sweets*** | 0.04 | 0.03 | 0.10 | 0.05 | - |
| **Supplements and meal replacements** | **0.18** | **-** | **0.01** | **0.22** | **0.12** |
| **Ready-to-eat-meals** | **4.22** | **5.80** | **6.98** | **4.22** | **1.47** |
| **Vegetables** | **11.14** | **8.39** | **8.65** | **11.23** | **13.27** |

**Table S8. Dietary sources of vitamin D (%) from food groups/subgroups by sex and age groups in the ANIBES Spanish population.**

| **VITAMIN D** | **Total 9–75** | **Children 9–12** | **Adolescents 13–17** | **Adults 18–64** | **Elderly 65–75** |
| --- | --- | --- | --- | --- | --- |
| **(%)** | **2009** | **213** | **211** | **1655** | **206** |
| **Alcoholic beverages** | **-** | **-** | **-** | **-** | **-** |
| ***High alcohol content beverages*** | - | - | - | - | - |
| ***Low alcohol content beverages*** | - | - | - | - | - |
| **Appetizers** | **-** | **-** | **-** | **-** | **-** |
| **Cereals/Grains** | **14.90** | **19.28** | **22.95** | **14.57** | **11.69** |
| ***Grains and flours*** | 0.36 | 0.51 | 0.50 | 0.38 | 0.20 |
| ***Breakfast cereals and cereal bars*** | 5.40 | 10.14 | 11.12 | 4.94 | 4.43 |
| ***Bread*** | - | - | - | - | - |
| *White bread* | - | - | - | - | - |
| *Brown bread* | - | - | - | - | - |
| *Others breads* | - | - | - | - | - |
| ***Pasta*** | 0.15 | 0.09 | 0.12 | 0.19 | - |
| ***Bakery and pastry*** | 9.00 | 8.54 | 11.22 | 9.05 | 7.06 |
| **Eggs** | **24.55** | **25.83** | **23.95** | **23.99** | **29.20** |
| **Fish** | **25.55** | **14.87** | **15.72** | **26.41** | **28.20** |
| ***White fish*** | 2.07 | 1.35 | 0.95 | 2.08 | 3.23 |
| ***Oily fish*** | 7.38 | 4.83 | 3.51 | 7.34 | 10.16 |
| ***Shellfish*** | 0.02 | 0.01 | - | 0.02 | - |
| ***Canned fish*** | 16.08 | 8.68 | 11.27 | 16.97 | 14.81 |
| **Fruits** | - | - | - | - | - |
| **Meat and meat products** | **1.90** | **1.23** | **1.40** | **1.95** | **1.42** |
| ***Meat*** | 0.65 | 0.21 | 0.40 | 0.64 | 0.70 |
| *Red meat* | 0.15 | 0.17 | 0.02 | 0.11 | 0.50 |
| *White meat* | 0.50 | 0.04 | 0.39 | 0.54 | 0.20 |
| *Poultry* | - | - | - | - | - |
| ***Viscera and offal*** | 0.37 | 0.19 | - | 0.41 | 0.27 |
| ***Sausages and other meat products*** | 0.89 | 0.82 | 1.00 | 0.90 | 0.45 |
| **Milk and dairy products** | **22.55** | **28.49** | **22.86** | **22.38** | **22.26** |
| ***Milk*** | 4.78 | 7.68 | 6.27 | 4.58 | 4.60 |
| *Whole milk* | 4.55 | 7.60 | 6.21 | 4.32 | 4.49 |
| *Semi skimmed milk* | - | - | - | - | - |
| *Skimmed milk* | - | - | - | - | - |
| *Other dairy* | 0.23 | 0.08 | 0.06 | 0.26 | 0.11 |
| ***Yogurt and fermented milk*** | 5.37 | 4.75 | 3.38 | 5.05 | 9.47 |
| *Skimmed Fermented milk* | 0.34 | 0.68 | 0.49 | 0.26 | 0.76 |
| *Whole fermented milk* | 0.58 | 0.17 | 0.04 | 0.58 | 1.04 |
| *Skimmed milk yogurts* | 0.38 | 0.06 | 0.17 | 0.32 | 0.89 |
| *Whole milk yogurt* | 4.07 | 3.85 | 2.67 | 3.88 | 6.78 |
| ***Cheese*** | 9.15 | 8.56 | 8.35 | 9.52 | 6.63 |
| ***Other dairy products*** | 3.25 | 7.50 | 4.87 | 3.24 | 1.56 |
| **Non-alcoholic beverages** | **-** | **-** | **-** | **-** | **-** |
| ***Water*** | - | - | - | - | - |
| ***Coffee and herbal teas*** | - | - | - | - | - |
| ***Sugared soft drinks*** | - | - | - | - | - |
| ***Unsweetened soft drinks*** | - | - | - | - | - |
| ***Sports drinks*** | - | - | - | - | - |
| ***Juices and nectars*** | - | - | - | - | - |
| ***Energy drinks*** | - | - | - | - | - |
| ***Other non-alcoholic beverages*** | - | - | - | - | - |
| **Oils and fats** | **2.98** | **2.29** | **3.29** | **2.96** | **3.54** |
| ***Olive oil*** | - | - | - | - | - |
| ***Other oils*** | - | - | - | - | - |
| ***Butter, margarine and shortening*** | 2.98 | 2.29 | 3.29 | 2.96 | 3.54 |
| **Pulses** | - | - | - | - | - |
| **Sauces and condiments** | **2.54** | **1.78** | **3.16** | **2.70** | **1.06** |
| **Sugars and sweets** | **0.06** | **0.03** | **0.24** | **0.04** | **-** |
| ***Sugar*** | - | - | - | - | - |
| ***Chocolate*** | 0.06 | 0.03 | 0.24 | 0.03 | - |
| ***Jams and other*** | - | - | - | - | - |
| ***Other sweets*** | - | - | - | - | - |
| **Supplements and meal replacements** | **0.25** | **0.39** | **-** | **0.28** | **0.63** |
| **Ready-to-eat-meals** | **4.72** | **5.83** | **6.43** | **4.73** | **2.00** |
| **Vegetables** | - | - | - | - | - |
